# Plasma Fatty Acid Composition Is Associated with Histological Findings of Nonalcoholic Steatohepatitis

**DOI:** 10.3390/biomedicines10102540

**Published:** 2022-10-12

**Authors:** Teruki Miyake, Shinya Furukawa, Bunzo Matsuura, Osamu Yoshida, Masumi Miyazaki, Akihito Shiomi, Sayaka Kanzaki, Hironobu Nakaguchi, Kotaro Sunago, Yoshiko Nakamura, Yusuke Imai, Takao Watanabe, Yasunori Yamamoto, Yohei Koizumi, Yoshio Tokumoto, Masashi Hirooka, Teru Kumagi, Masanori Abe, Yoichi Hiasa

**Affiliations:** 1 Department of Gastroenterology and Metabology, Ehime University Graduate School of Medicine, 454 Shitsukawa, Toon 791-0295, Ehime, Japan; 2 Health Services Center, Ehime University, Bunkyo, Matsuyama 790-8577, Ehime, Japan; 3 Department of Lifestyle-related Medicine and Endocrinology, Ehime University Graduate School of Medicine, 454 Shitsukawa, Toon 791-0295, Ehime, Japan; 4 Postgraduate Medical Education Center, Ehime University Graduate School of Medicine, 454 Shitsukawa, Toon 791-0295, Ehime, Japan

**Keywords:** plasma, fatty acids, fatty liver disease, histology, fibrosis, nonalcoholic steatohepatitis, nonalcoholic fatty liver disease

## Abstract

The relationship between advanced nonalcoholic steatohepatitis (NASH) and plasma fatty acid composition remains unknown. We aimed to examine the plasma fatty acid composition in biopsy-confirmed nonalcoholic fatty liver disease (NAFLD) and evaluate the relationship between histological findings and fatty acid composition. Overall, 235 patients (134 women) with NAFLD were enrolled. Comprehensive blood chemistry tests and histological examinations of liver samples were conducted. Multivariate analyses adjusted for age, sex, body mass index, alanine aminotransferase, hemoglobin A1c, creatinine, total cholesterol, triglyceride, and NAFLD Activity Score values showed that lower levels of arachidic, behenic, α-linolenic, eicosatetraenoic, docosapentaenoic, and docosahexaenoic acids and higher levels of mead acid were associated with fibrosis stage 3–4. Furthermore, higher lauric acid, myristic acid, and palmitic acid levels and monounsaturated fatty acids such as palmitoleic acid and oleic acid were significantly associated with high NAS in analyses adjusted for the same factors and fibrosis stage. The plasma fatty acid composition was associated with the histological evidence of NASH. Increased synthesis of fatty acids is associated with NASH; insufficient intake of n-3 essential fatty acids and reduced elongation of fatty acids are associated with fibrosis in NASH. These features may help clinicians to understand and treat advanced NASH cases.

## 1. Introduction

Unhealthy eating habits and lack of exercise are associated with increasing rates of nonalcoholic fatty liver disease (NAFLD). Many patients with NAFLD retain a normal quality of life [1]. However, some patients with NAFLD may progress to nonalcoholic steatohepatitis (NASH) and cirrhosis [2,3,4]. In addition, advanced NASH is a high-risk factor for liver failure and hepatocellular carcinoma (HCC) [5,6]. Therefore, it is important to diagnose advanced and progressing NASH and proactively intervene.

Fatty acids are the substrates of triglycerides and are important for fatty liver formation. Moreover, the fatty acid composition is associated with various diseases. The higher palmitic acid (16:0), palmitoleic acid (16:1n–7), and stearic acid (18:0) levels in plasma and plasma phospholipids (PL) have been associated with insulin resistance and diabetes [7,8,9]. In contrast, higher very-long-chain saturated fatty acid levels in plasma phospholipids, such as arachidic acid (20:0), behenic acid (22:0), and lignoceric acid (24:0), have been associated with lower risks of incident diabetes mellitus [10,11] and carotid artery plaques [12]. In addition, a previous meta-analysis has shown that α-linolenic acid (18:3n3), eicosatetraenoic acid (20:5n3), docosapentaenoic acid (22:5n3), and docosahexaenoic acid (22:6n3) decreased the risk of type 2 diabetes mellitus, cardiovascular disease, colorectal cancer, or mortality [13].

In NAFLD, lipidomic analysis revealed that the changes to lipid moieties helped to distinguish NASH from nonalcoholic fatty liver (NAFL) and diagnose significant fibrosis [14,15,16,17]. However, the relationship between clinically problematic advanced NASH and fatty acids remains unknown. Herein, we aimed to examine the plasma fatty acid composition in biopsy-confirmed NAFLD, and to analyze the relationship between histological findings and fatty acid composition.

## 2. Materials and Methods

### 2.1. Patients

This cross-sectional study enrolled 240 patients (136 women), aged 20–79 years, with NAFLD, diagnosed by liver biopsy at Ehime University Hospital from February 2011 to March 2021. The inclusion criteria were as follows: (1) diagnosis of fatty liver by histological examination for elevated liver enzyme or ultrasonography, computed tomography, and magnetic resonance imaging findings indicative of liver injury; (2) moderate alcohol consumption habits (men, <30 g/day; women, <20 g/day); (3) absence of viral hepatitis, autoimmune hepatitis, drug-induced liver disease, primary biliary cholangitis, primary sclerosing cholangitis, hemochromatosis, Wilson’s disease, and a-1-antitrypsin deficiency-associated liver disease; and (4) absence of decompensated liver failure or HCC.

After written informed consent was obtained, patient data were assigned numerical codes to preserve anonymity throughout the study. All data were kept in a secure database and analyzed. The data of five individuals were excluded according to the following exclusion criteria: (1) incomplete data (*n* = 2), (2) steroid treatment (*n* = 1), (3) cancer or other complications (*n* = 1), and/or (4) unsuitability for the study (*n* = 1). Finally, data from 235 patients (134 women) were included in the analysis. The study protocol was approved by the Ethics Committee of Ehime University Hospital (Approval ID# 1012004, 1709008), and the study was conducted according to the principles of the Declaration of Helsinki.

### 2.2. Measurements

Medical history, physical examination, and biochemical examination findings were examined. Medical history interrogated factors such as age, sex, case history, drinking habits, and history of prescribed medication. Patients were wearing light gowns and no shoes when height and body were measured. Venous blood samples were collected on the morning of the second day of hospitalization after a 12-h fast. Plasma fatty acid concentrations were measured by gas chromatography. Plasma was mixed with derivatizing reagent (KOKUSAN CHEMICAL Co., Ltd. Tokyo, Japan) and internal standard liquid, and was stirred to obtain methyl-esterified specimens. Then, NaOH and n-hexane were added to each methyl-esterified sample. The upper layer of the sample was separated after thorough mixing and centrifugation, and was measured using a gas chromatograph (GC-17A and GC-2010, SHIMADZU CORPORATION Co. Ltd., Kyoto, Japan) in SRL, Inc. (Tokyo, Japan). The biochemical data included alanine aminotransferase (ALT), gamma-glutamyl transpeptidase, creatinine (Cre), hemoglobin A1c (HbA1c), total cholesterol (TC), and triglyceride (TG) levels.

### 2.3. Histological Evaluation

**Figure 1:**
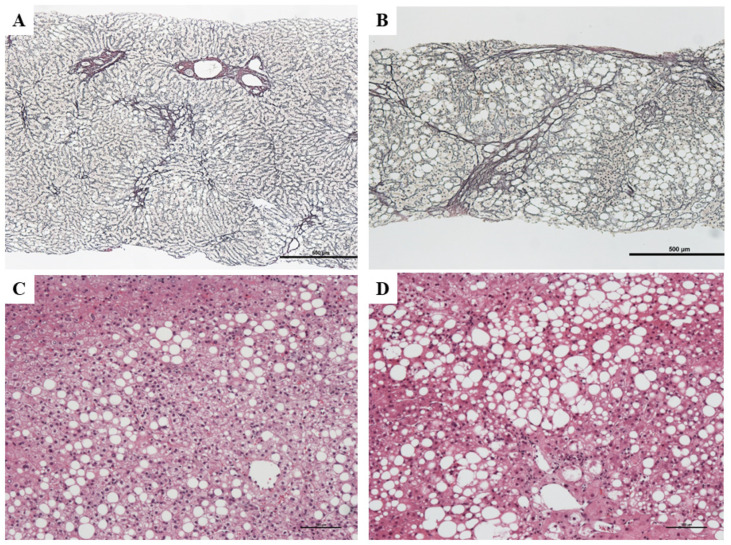
Histological findings of NAFLD. (**A**) Tissue sample with fibrosis stage 1b. (**B**) Tissue sample with fibrosis stage 3. (**C**) Tissue sample with NAS 2: steatosis, 1; lobular inflammation, 1; and ballooning degeneration, 2. (**D**) Tissue sample with NAS 6: steatosis, 2; lobular inflammation, 2; and ballooning degeneration, 2.

Liver tissue samples were collected by percutaneous liver biopsy under ultrasonic or laparoscopic guidance and were embedded in paraffin. The liver specimens were stained with hematoxylin and eosin and reticulin silver stain. Two hepatopathologists (Takao Watabnabe and Osamu Yoshida), blinded to patient clinical information, assessed the liver specimens. A liver specimen 1.5 cm in length, and/or with at least six portal tracts, was defined as adequate. The evaluation of liver tissue was performed according to the NAFLD Activity Score (NAS); fibrosis stage was evaluated as proposed by Kleiner et al. (Figure 1) [18,19]. The NAS consists of a numerical grade for steatosis (0–3), lobular inflammation (0–3), and ballooning degeneration, and its final value is the sum of the three grades [18,19]. Fibrosis stage was determined according to the following criteria: stage 0, absence of fibrosis; stage 1a, delicate perisinusoidal fibrosis; stage 1b, dense perisinusoidal fibrosis; stage 1c, portal-only fibrosis without perisinusoidal fibrosis; stage 2, combined perisinusoidal and portal/periportal fibrosis; stage 3, bridging fibrosis; and stage 4, cirrhosis [18,19]. Patients with stage 3–4 were determined to have advanced fibrosis, while NAS 5–8 was defined as a high NAS.

### 2.4. Statistical Analysis

Statistical analyses were performed using the JMP software, version 14.2 (SAS Institute, Cary, NC, USA). Since the continuous variables, such as behenic and lignoceric acids, proved to be normally distributed, they were analyzed using an unpaired *t*-test and the Tukey–Kramer test. Conversely, other fatty acids, which were non-normally distributed, were analyzed using the Wilcoxon test and Steel–Dwass test. Spearman’s correlation coefficients were used to assess the correlation between the fatty acids and the clinical variables. Crude odds ratios (ORs) and their 95% confidence intervals for the association between histological findings and fatty acid plasma levels were derived in logistic regression analysis. Multiple logistic regression analysis was adjusted for age, sex, BMI, ALT, HbA1c, Cre, TC, TG, and NAS values or fibrosis stage, which were selected a priori as potential confounding factors. Data are shown as medians (IQR) or as counts (percentages). *p*-values of <0.05 were considered statistically significant.

## 3. Results

### 3.1. Patient Characteristics

The patients’ characteristics are presented in Table 1.

Patients with advanced fibrosis were significantly older, more obese, and had higher GGT and HbA1c levels and lower TC levels compared to patients with non-advanced fibrosis (Table 2). Conversely, patients with a high NAS reflected a higher percentage of men and had higher GGT, HbA1c, and TG levels compared to patients with a low NAS (Table 3).

Table 4 demonstrates the correlations between the fatty acids and clinical variables. AST and GGT were not found to be strongly correlated with each fatty acid; however, the TC level was strongly correlated with those of the behenic and lignoceric acids, while the TG level was strongly correlated with those of the myristic, palmitic, stearic, and oleic acids (Table 4).

### 3.2. Plasma Fatty Acid Compositions in NAFLD

The comparisons of fatty acid levels between non-advanced fibrosis stage and advanced fibrosis stage or between low and high NAS groups are shown in Table 2 and Table 3. The levels of saturated fatty acids, such as arachidic acid, behenic acid, and lignoceric acid, and polyunsaturated fatty acids, such as linoleic acid, α-linolenic acid, arachidonic acid, eicosatetraenoic acid, docosapentaenoic acid, and docosahexaenoic acid, were lower in patients with fibrosis stage 3–4 than in those with fibrosis stage 0–2 (Table 5). Meanwhile, the levels of saturated fatty acids, such as lauric acid, and polyunsaturated fatty acids, such as mead acid, were higher in patients with fibrosis stage 3–4 than in those with fibrosis stage 0–2 (Table 5). The levels of saturated fatty acids (lauric, myristic, palmitic, stearic, and behenic acids), monounsaturated fatty acids (myristoleic, palmitoleic, oleic, eicosadienoic, and erucic acids), and polyunsaturated fatty acids (linoleic, α-linolenic, mead, dihomo-γ-linolenic, arachidonic, adrenic, docosapentaenoic, and docosahexaenoic acids) were higher in patients with NAS values of 5–8 than in those with NAS values of 1–4 (Table 6). Meanwhile, the levels of monounsaturated fatty acids, such as eicosanoid acid, were lower in patients with NAS values of 5–8 than in those with NAS values of 1–4 (Table 6).

Table 7, Table 8 and Table 9 demonstrate the composition of the fatty acids with regard to the three constituents of the NAS. The levels of saturated fatty acids, monounsaturated fatty acids (with the exception of erucic and nervonic acids), and polyunsaturated fatty acids (with the exception of eicosapentaenoic acid) increased as the degree of steatosis worsened (Table 7). The levels of saturated fatty acids (lauric, myristic, and palmitic acids), monounsaturated fatty acids (palmitoleic and oleic acids), and polyunsaturated fatty acids (eicosadienoic, di-homo-γ-linolenic, and adrenic acids) increased with increased lobular inflammation scores (Table 8). On ballooning, the high levels of saturated fatty acids, such as lauric acid, myristic acid, palmitic acid, and stearic acid, monounsaturated fatty acids, such as myristoleic acid, palmitoleic acid, and oleic acid, and polyunsaturated fatty acids, such as eicosadienoic acid, mead acid, dihomo-γ-linolenic acid, and adrenic acid, showed high scores (Table 9).

### 3.3. Relationship between Fatty Acid Levels and Histological Findings

**Figure 2:**
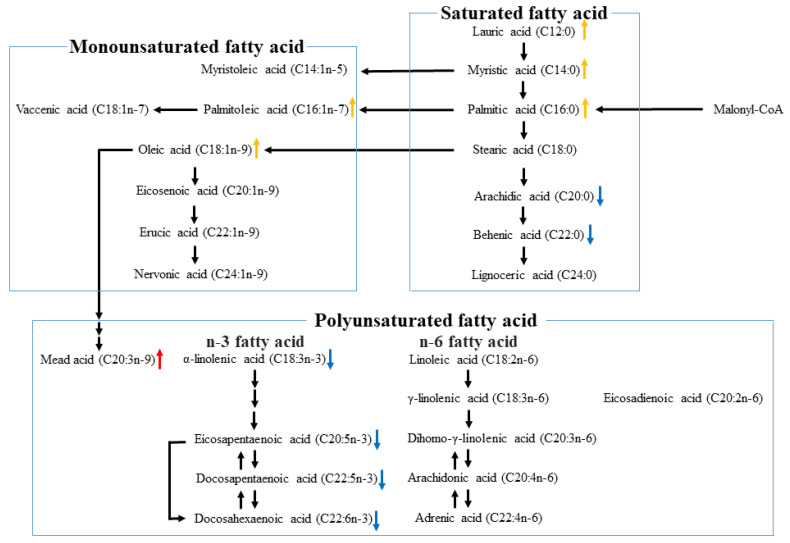
The relationships between fatty acid levels, the metabolic pathway, and NASH histological findings. ↑ indicates elevation in fatty acid levels in advanced fibrosis (Stage 3–4). ↓ indicates reduction in fatty acid levels in advanced fibrosis (Stage 3–4). ↑ indicates elevation in fatty acid levels in high NAS (NAS 5–8).

Lower levels of saturated fatty acids, such as arachidic acid, behenic acid, and lignoceric acid, and polyunsaturated fatty acids, such as linoleic acid, α-linolenic acid, arachidonic acid, eicosapentaenoic acid, docosapentaenoic acid, and docosahexaenoic acid, and higher levels of mead acid were significantly associated with fibrosis stage 3–4 (Table 10). In addition, after adjustments for age, sex, BMI, ALT, HbA1c, Cre, TC, TG, and NAS values, significant associations remained among lower levels of arachidic acid, behenic acid, α-linolenic acid, eicosapentaenoic acid, docosapentaenoic acid, and docosahexaenoic acid, and higher levels of mead acid and fibrosis stage 3–4 (Table 10, Figure 2).

Concurrently, saturated fatty acids, such as lauric acid, myristic acid, palmitic acid, stearic acid, and arachidic acid, monounsaturated fatty acids, such as myristoleic acid, palmitoleic acid, oleic acid, and eicosenoic acid, and polyunsaturated fatty acids, such as linoleic acid, α-linolenic acid, eicosadienoic acid, mead acid, arachidonic acid, adrenic acid, and docosapentaenoic acid were associated with high NAS values (Table 11). Multivariate analysis adjusted for age, sex, BMI, ALT, HbA1c, Cre, TC, and TG values and fibrosis stage showed that higher levels of lauric acid, myristic acid, and palmitic acid and monounsaturated fatty acids, such as palmitoleic acid and oleic acid, were significantly associated with high NAS values (Table 11, Figure 2).

## 4. Discussion

This study showed that very-long-chain fatty acids and n-3 polyunsaturated fatty acid levels were decreased and that mead acid levels associated with essential fatty acid shortages [20] were increased in NASH patients with advanced fibrosis. Concurrently, patients with high NAS values had high levels of long-chain saturated fatty acids and monounsaturated fatty acids. These results suggest that the increased synthesis of fatty acids might be involved in the activity of NASH and that insufficient intake of n-3 essential fatty acids and reduced elongation of fatty acids might contribute to the development of fibrosis in NASH. These features may be useful for identifying advanced and highly active NASH and helping to develop treatments.

Previous studies have reported on the association between NAFLD and fatty acid fraction. Araya et al. enrolled 11 control participants, 10 patients with NAFL, and nine patients with NASH to examine the levels of polyunsaturated fatty acid in total liver lipids, TG, and phospholipids, and revealed that the n-6:n-3 ratio (weight/weight) in liver total lipids in the NASH group was higher than those in the control and NAFL groups [21]. Puri et al. analyzed the free fatty acid, diacylglycerol, triacylglycerol, free cholesterol, cholesterol ester, and phospholipid content in the liver, and the distribution of fatty liver, in these classes, among nine control, NAFL, and NASH (fibrosis stage 0–2) participants, respectively. These authors showed that the n-6:n-3 ratio (mol %/ mol %) of free fatty acids was higher in the NASH group than in the NAFL group [22]. Although these reports showed that the n-6/n-3 ratio of fatty acid composition in the liver helps to distinguish between NAFL and NASH, the obtained results were not adjusted for confounding factors. In addition, the effect of fatty acid composition on the activity and fibrosis of NASH was not examined. Yamada et al. measured the fatty acid composition in the livers of 63 patients with NAFL and 43 patients with NASH (fibrosis stage 0/ 1/ 2/ 3/ 4, N = 8/ 67/ 15/ 7/ 6) and examined the relationship between fatty acid composition and histological findings and enzyme gene expression involved in fatty acid synthesis and degradation [23]. In the liver, patients with NASH had higher palmitoleic acid (C16:1n-7) levels, a lower stearic acid (C18:0)/palmitic acid (C16:0) ratio (weight/weight), and a higher palmitoleic acid (C16:1n-7)/palmitic acid (C16:0) ratio (weight/weight), higher oleic acid (C18:1n-9)/stearic acid (C18:0) ratio (weight/weight), and higher n-6/n-3 ratio (weight/weight), as well as higher expression of fatty acid metabolism-related genes, including SCD1, ELOVL6, SREBP1, FAS, and PPARγ, than patients with simple steatosis [15]. These results are consistent with our results obtained in the plasma. However, this study did not examine the association with advanced fibrosis or activity. Fridén et al. included 68 NAFLD patients (fibrosis stage 0/1/2/3/4, N = 4/32/19/3/2, respectively) and examined fatty acid levels and fatty acid ratios in three lipid fractions (cholesteryl esters, TGs, and phospholipids) in the liver and plasma; comparisons were made between patients with fibrosis stage 0–1 and those with fibrosis stage 2–4 [17]. The authors showed that fibrosis stage 2–4 was positively associated with behenic acid (22:0) (%) of phospholipids (liver) and inversely associated with behenic acid (22:0) of phospholipids (plasma), docosahexaenoic acid (22:6n3) (%) of phospholipids (liver), and oleic acid (18:1n9) (%) of TG (liver and plasma) [17]. In addition, fibrosis stage 2–4 was positively associated with the SFA of phospholipids (liver and plasma) and inversely associated with polyunsaturated fatty acid (%) of phospholipids (liver) and monounsaturated fatty acid (%) of TG (liver and plasma) [17]. Although this study examined the fatty acid portion of three lipid fractions in the liver and plasma, the association between plasma fatty acid fraction and advanced fibrosis and activity in NASH, which was our primary outcome, was unknown. Concurrently, Allard et al. examined the fatty acid composition in the livers of 18 patients with NAFL, 38 patients with NASH, and 17 patients with minimal findings (histological normal) [23]. Total n-6 polyunsaturated fatty acid (%) was decreased in patients with NASH, and there was no significant difference in the n-6/ n-3 ratio (%/%) between NAFL and NASH patients [23]. Although these results differ from previous reports, they were not adjusted for confounding factors.

The changes in fatty acid composition may be involved in the pathogenesis of NASH. Our study showed that the levels of plasma very-long-chain saturated fatty acids, such as arachidic acid (20:0) and behenic acid (22:0), and polyunsaturated fatty acids, such as α-linolenic acid (18:3n3), eicosapentaenoic acid (20:5n3), docosapentaenoic acid (22:5n3), and docosahexaenoic acid (22:6n3), were decreased in advanced fibrosis, while the levels of plasma lauric acid (12:0), myristic acid (14:0), palmitic acid (16:0), palmitoleic acid (16:1n7), and oleic acid (18:1n9) were increased in patients with high NAS. Increased levels of plasma saturated fatty acids, such as palmitic acid (16:0) and stearic acid (18:0), and monounsaturated fatty acids, such as palmitoleic acid (16:1n–7), are associated with increased insulin resistance and diabetes risk [7,8,9,24]; however, increased plasma levels of saturated very-long-chain fatty acids, such as arachidic acid (20:0), behenic acid (22:0), and lignoceric acid (24:0), and n-3 polyunsaturated fatty acids, such as α-linolenic acid (18:3n3), eicosapentaenoic acid (20:5n3), docosapentaenoic acid (22:5n3), and docosahexaenoic acid (22:6n3), may decrease this risk [10,11,13]. Therefore, the change in plasma fatty acid levels in this study might be associated with the deterioration of glucose metabolism. Diabetes is a risk factor for the progression of NASH and may increase the risk of progression to fibrosis. In addition, an increase in plasma palmitic acid level and a decrease in plasma very-long-chain saturated fatty acids may reflect an unhealthy lifestyle, such as high red and processed meat consumption and little physical activity [25]. An unhealthy lifestyle may affect the progression of NASH. On the other hand, an increase in plasma monounsaturated fatty acid levels reflects the activation of SCD-1, which exacerbates fat deposition in the liver [26]. Moreover, polyunsaturated fatty acids suppress SREBP-1c, SCD-1, and fatty acid oxidation; a decrease in n-3 polyunsaturated fatty acid level might exacerbate NAFLD [27,28,29].

The strength of this study is the relatively large sample size and inclusion of patients with advanced fibrosis that is clinically problematic. However, this study has some limitations. First, the fatty acid composition of lipids in the liver remains unclear. However, plasma fatty acid composition in CE, TG, and PL reflects the composition of TG in the liver [16]. The plasma fatty acid fraction should be examined to understand the fatty acid metabolism in the liver. Second, some participants were taking hypoglycemic or lipid-lowering medications, but the effects of these medications were unaccounted for. Finally, this study was cross-sectional; the mechanism of the fatty acid composition’s impact on the development of NASH remains unclear.

In conclusion, this study has shown that the plasma fatty acid composition was associated with histological findings of NASH. These features may be useful for identifying advanced and highly active NASH. Furthermore, affecting the enzymes associated with the increased synthesis of fatty acids or the intake of deficient fatty acids could improve NAFLD pathogenesis and potentially lead to the development of treatment options for NAFLD.

## Figures and Tables

**Table 1:** Patients’ characteristics.

Age, Year	59 (46–67)
Sex, N (Men/Women)	101/134
BMI, kg/m^2^	27.4 (24.1–30.3)
ALT, U/L	63 (37–102)
GGT, U/L	62 (37–113)
Cre, mg/dL	0.68 (0.57–0.85)
HbA1c, %	6.2 (5.7–7.2)
TC, mg/dL	194 (168–220)
TG, mg/dL	130 (92–173)
Stage (0/1a,1b,1c/2/3/4), N	(52/55/33/35/60)
NAS (0/1/2/3/4/5/6/7/8), N	(0/18/19/35/40/47/35/29/12)
Antidiabetic agents, N	
Biguanide	46
Dipeptidyl peptidase-4 inhibitor	36
Sulfonylurea	21
Alpha-glucosidase inhibitor	8
Pioglitazone	4
Glinide	5
Glucagon like peptide-1receptor agonist	6
Sodium-glucose transport protein 2 inhibitor	6
Insulin	19
Lipid-lowering agents, N	
Statin	55
Ezetimibe	3
Colestilan	0
Fibrate	6
Eicosapentaenoic acid ethyl ester	7
Saturated fatty acid, μg/mL	
Lauric acid (12:0)	1.7 (1.1–2.7)
Myristic acid (14:0)	25.3 (19–34.9)
Palmitic acid (16:0)	767.7 (654.2–914)
Stearic acid (18:0)	225.6 (192.6–258.2)
Arachidic acid (20:0)	7.8 (6.6–8.9)
Behenic acid (22:0)	19.2 (16.5–21.9)
Lignoceric acid (24:0)	16.5 (14.2–19.3)
Monounsaturated fatty acid, μg/mL	
Myristoleic acid (14:1n5)	1.1 (0.1–1.8)
Palmitoleic acid (16:1n7)	77.8 (59.2–106.9)
Oleic acid (18:1n9)	714.8 (599.5–887.8)
Eicosenoic acid (20:1n9)	5.0 (4.1–6.4)
Erucic acid (22:1n9)	1.2 (1.0–1.6)
Nervonic acid (24:1n9)	36.1 (31.5–41.3)
Polyunsaturated fatty acid, μg/mL	
Linoleic acid (18:2n6)	814.9 (697.6–950.7)
γ-linolenic acid (18:3n6)	10.3 (7.4–14.4)
α-linolenic acid (18:3n3)	21.7 (17.2–28.6)
Eicosadienoic acid (20:2n6)	6.5 (5.5–7.8)
Mead acid (20:3n9)	2.6 (1.7–3.9)
Dihomo-γ-linolenic acid (20:3n6)	48.4 (36.9–60.2)
Arachidonic acid (20:4n6)	196.4 (168.4–234.0)
Eicosapentaenoic acid (20:5n3)	47.8 (29.4–70.0)
Adrenic acid (22:4n6)	5.7 (4.5–7.2)
Docosapentaenoic acid (22:5n3)	19.8 (16.2–24.8)
Docosahexaenoic acid (22:6n3)	138.5 (112.1–171.9)

Data are presented as median (interquartile range) or as numbers. BMI, body mass index; ALT, alanine aminotransferase; GGT, gamma-glutamyl transpeptidase; Cre, creatinine; HbA1c, hemoglobin A1c; TC, total cholesterol; TG, triglycerides; NAS, NAFLD Activity Score.

**Table 2:** Patients’ characteristics (non-advanced fibrosis stage vs. advanced fibrosis stage).

	Stage 0–2 (*n* = 140)	Stage 3–4 (*n* = 95)	*p*-Value
Age, year	54 (40.3–64)	62 (55–69)	<0.01
Sex, N (Men/Women)	66/74	35/60	0.12
BMI, kg/m^2^	26.4 (23.8–29.7)	28.7 (25.0–31.4)	0.01
ALT, U/L	60.5 (36.3–102)	65 (37–103)	0.64
GGT, U/L	51 (30.3–111.8)	78 (49–118)	<0.01
Cre, mg/dL	0.71 (0.56–0.85)	0.66 (0.59–0.85)	0.49
HbA1c, %	6.0 (5.6–6.8)	6.6 (6.0–7.7)	<0.01
TC, mg/dL	196 (173.3–223)	191 (161–211)	<0.01
TG, mg/dL	132.5 (97.5–172.8)	126 (88–178)	0.26

Data are presented as median (interquartile range) or as numbers. *p*-values were determined using the unpaired *t*-test or Wilcoxon test. *p* < 0.05 was considered statistically significant. BMI, body mass index; ALT, alanine aminotransferase; GGT, gamma-glutamyl transpeptidase; Cre, creatinine; HbA1c, hemoglobin A1c; TC, total cholesterol; TG, triglycerides.

**Table 3:** Characteristics (low NAS vs. high NAS).

	NAS 1–4 (*n* = 112)	NAS 5–8 (*n* = 123)	*p*-Value
Age, year	60 (46–69)	58 (46–65)	0.49
Sex, N (Men/Women)	56/56	45/78	0.04
BMI, kg/m^2^	25.6 (23.1–29.3)	28.3 (25.2–31.4)	0.78
ALT, U/L	38 (29–62.5)	88 (61–124)	0.68
GGT, U/L	45 (28–108)	73 (48–118)	<0.01
Cre, mg/dL	0.74 (0.6–0.86)	0.65 (0.55–0.84)	0.10
HbA1c, %	6.1 (5.6–6.8)	6.4 (5.9–7.6)	<0.01
TC, mg/dL	189 (164–212)	198 (177–220)	0.06
TG, mg/dL	122 (85–153)	144 (101–192)	<0.01

Data are presented as median (interquartile range) or as numbers. *p*-values were determined using unpaired *t*-test or the Wilcoxon test. *p* < 0.05 was considered statistically significant. BMI, body mass index; ALT, alanine aminotransferase; GGT, gamma-glutamyl transpeptidase; Cre, creatinine; HbA1c, hemoglobin A1c; TC, total cholesterol; TG, triglycerides; NAS, NAFLD Activity Score.

**Table 4:** Correlations between fatty acids and clinical variables.

	ALT *r*	*p*-Value	GGT *r*	*p*-Value	TC *r*	*p*-Value	TG *r*	*p*-Value
Saturated fatty acid								
Lauric acid (12:0)	0.16	0.01	0.14	0.03	0.20	<0.01	0.62	<0.01
Myristic acid (14:0)	0.27	<0.01	0.28	<0.01	0.33	<0.01	0.84	<0.01
Palmitic acid (16:0)	0.33	<0.01	0.28	<0.01	0.46	<0.01	0.87	<0.01
Stearic acid (18:0)	0.37	<0.01	0.34	<0.01	0.56	<0.01	0.76	<0.01
Arachidic acid (20:0)	0.23	<0.01	0.09	0.18	0.66	<0.01	0.27	<0.01
Behenic acid (22:0)	0.20	<0.01	0.08	0.21	0.74	<0.01	0.21	<0.01
Lignoceric acid (24:0)	0.13	0.045	0.06	0.39	0.71	<0.01	0.13	0.04
Monounsaturated fatty acid								
Myristoleic acid (14:1n5)	0.07	0.31	0.15	0.02	0.04	0.53	0.37	<0.01
Palmitoleic acid (16:1n7)	0.22	<0.01	0.23	<0.01	0,29	<0.01	0.64	<0.01
Oleic acid (18:1n9)	0.29	<0.01	0.25	<0.01	0.35	<0.01	0.92	<0.01
Eicosenoic acid (20:1n9)	0.12	0.08	0.14	0.03	0.17	0.01	0.64	<0.01
Erucic acid (22:1n9)	0.01	0.85	0.02	0.75	0.30	<0.01	0.21	<0.01
Nervonic acid (24:1n9)	0.05	0.43	−0.02	0.75	0.58	<0.01	−0.14	0.04
Polyunsaturated fatty acid								
Linoleic acid (18:2n6)	0.21	<0.01	0.11	0.11	0.65	<0.01	0.58	<0.01
γ-linolenic acid (18:3n6)	0.14	0.04	0.24	<0.01	0.33	<0.01	0.50	<0.01
α-linolenic acid (18:3n3)	0.14	0.04	0.14	0.04	0.33	<0.01	0.76	<0.01
Eicosadienoic acid (20:2n6)	0.20	<0.01	0.25	<0.01	0.40	<0.01	0.65	<0.01
Mead acid (20:3n9)	0.20	<0.01	0.30	<0.01	0.18	<0.01	0.53	<0.01
Dihomo-γ-linolenic acid (20:3n6)	0.40	<0.01	0.29	<0.01	0.49	<0.01	0.55	<0.01
Arachidonic acid (20:4n6)	0.23	<0.01	0.21	<0.01	0.44	<0.01	0.37	<0.01
Eicosapentaenoic acid (20:5n3)	−0.09	0.17	0.001	0.99	0.19	<0.01	0.08	0.23
Adrenic acid (22:4n6)	0.28	<0.01	0.28	<0.01	0.31	<0.01	0.63	<0.01
Docosapentaenoic acid (22:5n3)	0.16	0.01	0.20	<0.01	0.37	<0.01	0.53	<0.01
Docosahexaenoic acid (22:6n3)	0.04	0.51	0.04	0.57	0.30	<0.01	0.30	<0.01

Spearman’s correlation coefficients were used to assess the correlation between the fatty acids and the clinical variables. *p* < 0.05 was considered statistically significant. ALT, alanine aminotransferase; GGT, gamma-glutamyl transpeptidase; TC, total cholesterol; TG, triglycerides.

**Table 5:** Plasma fatty acid composition (μg/mL) (non-advanced fibrosis stage vs. advanced fibrosis stage).

	Stage 0–2 (*n* = 140)	Stage 3–4 (*n* = 95)	*p*-Value
Saturated fatty acid			
Lauric acid (12:0)	1.5 (1.1–2.5)	2.0 (1.2–2.8)	0.03
Myristic acid (14:0)	25.4 (19.1–34.5)	24.8 (18.1–35.3)	0.78
Palmitic acid (16:0)	767.4 (660.7–930.9)	767.7 (650.4–891.5)	0.68
Stearic acid (18:0)	223.3 (191.6–268.8)	227.7 (193.0–251.4)	0.50
Arachidic acid (20:0)	8.3 (7.0–9.3)	7.4 (6.3–8.2)	<0.01
Behenic acid (22:0)	19.8 (17.2–22.5)	18.3 (15.1–20.7)	<0.01
Lignoceric acid (24:0)	16.9 (14.8–19.5)	15.6 (13.1–18.6)	<0.01
Monounsaturated fatty acid			
Myristoleic acid (14:1n5)	0.9 (0.1–1.7)	1.2 (0.1–2.0)	0.07
Palmitoleic acid (16:1n7)	78.1 (56.2–102.5)	77.2 (61.3–117.4)	0.21
Oleic acid (18:1n9)	718.4 (609.1–875.6)	705.4 (572.4–896.4)	0.74
Eicosenoic acid (20:1n9)	4.9 (4.0–6.2)	5.1 (4.3–6.5)	0.47
Erucic acid (22:1n9)	1.2 (1.0–1.6)	1.1 (1.0–1.5)	0.24
Nervonic acid (24:1n9)	37.2 (32.0–41.7)	34.2 (30.4–40.4)	0.05
Polyunsaturated fatty acid			
Linoleic acid (18:2n6)	828.4 (723.9–974.2)	781.0 (667.9–876.5)	0.02
γ-linolenic acid (18:3n6)	10.6 (7.5–15.3)	9.7 (7.2–13.4)	0.12
α-linolenic acid (18:3n3)	22.3 (18.6–29.5)	20.1 (15.7–27.5)	0.02
Eicosadienoic acid (20:2n6)	6.4 (5.4–7.7)	6.6 (5.5–7.9)	0.88
Mead acid (20:3n9)	2.5 (1.7–3.5)	3 (1.7–4.3)	0.04
Dihomo-γ-linolenic acid (20:3n6)	48.2 (38.1–60.1)	49 (34.5–60.3)	0.91
Arachidonic acid (20:4n6)	206.7 (176.7–241.1)	186.8 (152.9–221.9)	<0.01
Eicosapentaenoic acid (20:5n3)	56.1 (35.2–75.1)	40.5 (26.6–58.2)	<0.01
Adrenic acid (22:4n6)	5.6 (4.5–7.1)	5.8 (4.6–7.5)	0.40
Docosapentaenoic acid (22:5n3)	20.6 (17.0–25.1)	18.6 (15.4–23.5)	0.02
Docosahexaenoic acid (22:6n3)	150.1 (119.4–187.9)	123.2 (102.4–150.7)	<0.01

Data are presented as median (interquartile range) or as numbers. *p*-values were determined using unpaired *t*-test or the Wilcoxon test. *p* < 0.05 was considered statistically significant.

**Table 6:** Plasma fatty acid composition (μg/mL) (low NAS vs. high NAS).

	NAS 1–4 (*n* = 112)	NAS 5–8 (*n* = 123)	*p*-Value
Saturated fatty acid			
Lauric acid (12:0)	1.3 (0.9–2.1)	2.2 (1.3–3.1)	<0.01
Myristic acid (14:0)	20.5 (16.1–27.8)	29.8 (21.9–37.6)	<0.01
Palmitic acid (16:0)	705.0 (595.5–795.0)	842.3 (719.2–1002.9)	<0.01
Stearic acid (18:0)	208.6 (179.4–240.3)	241.4 (212.3–268.9)	<0.01
Arachidic acid (20:0)	7.6 (6.3–8.8)	7.9 (6.9–8.9)	0.05
Behenic acid (22:0)	18.6 (16.0–21.8)	19.8 (17.0–22.4)	0.04
Lignoceric acid (24:0)	16.5 (14.4–19.1)	16.6 (14.1–19.4)	0.22
Monounsaturated fatty acid			
Myristoleic acid (14:1n5)	0.8 (0.1–1.5)	1.4 (0.1–2.2)	<0.01
Palmitoleic acid (16:1n7)	64.9 (51.3–87.9)	90.1 (71.1–123.1)	<0.01
Oleic acid (18:1n9)	653.1 (543.4–785.6)	776.1 (662.0–963.3)	<0.01
Eicosenoic acid (20:1n9)	5.5 (4.6–6.5)	4.6 (3.8–6.0)	<0.01
Erucic acid (22:1n9)	1.1 (1.0–1.6)	1.3 (1.0–1.6)	0.03
Nervonic acid (24:1n9)	36.4 (31.2–41.1)	35.7 (31.7–41.7)	0.88
Polyunsaturated fatty acid			
Linoleic acid (18:2n6)	782.5 (672.4–882.8)	831.2 (728.6–994.1)	<0.01
γ-linolenic acid (18:3n6)	9.8 (6.6–13.9)	10.5 (8.2–15.3)	0.06
α-linolenic acid (18:3n3)	20.3 (15.8–25.9)	22.4 (18.5–29.8)	0.01
Eicosadienoic acid (20:2n6)	6.2 (5.1–7.4)	6.7 (5.7–8.1)	<0.01
Mead acid (20:3n9)	2.3 (1.4–3.3)	2.9 (2–4.2)	<0.01
Dihomo-γ-linolenic acid (20:3n6)	42.3 (33.2–50.6)	53.4 (44.5–64.8)	<0.01
Arachidonic acid (20:4n6)	186.8 (148.7–223.2)	211.1 (176.5–240.5)	0.01
Eicosapentaenoic acid (20:5n3)	48.0 (30.1–72.5)	47.8 (29.0–66.9)	0.76
Adrenic acid (22:4n6)	5.2 (4.0–6.4)	6.3 (4.9–7.8)	<0.01
Docosapentaenoic acid (22:5n3)	18.8 (15.1–23.2)	20.7 (17.7–26.1)	<0.01
Docosahexaenoic acid (22:6n3)	129.0 (108.7–170.4)	146.0 (114.7–176.9)	0.10

Data are presented as median (interquartile range) or as numbers. *p*-values were determined using unpaired *t*-test or the Wilcoxon test. *p* < 0.05 was considered statistically significant.

**Table 7:** Plasma fatty acid composition (μg/mL) (steatosis grade).

Steatosis Grade	<33% (*n* = 88)	33–66% (*n* = 78)	>66% (*n* = 69)	*p*-Value
Saturated fatty acid				
Lauric acid (12:0)	1.4 (0.9–2.25)	1.7 (1.1–2.8)	2.2 (1.4–3.1)	<0.05 ^†^
Myristic acid (14:0)	21.0 (17.2–27.5)	25.5 (18.3–37.7)	31.3 (23.5–39.9	<0.05 ^†^
Palmitic acid (16:0)	702.1 (607.7–783.3)	765.4 (635.2–933.4)	882.9 (757.8–1030.6)	<0.05 *^,†,‡^
Stearic acid (18:0)	208.2 (179.4–241.3)	220.5 (192.9–252.8)	249.5 (219.9–278.0)	<0.05 ^†,‡^
Arachidic acid (20:0)	7.3 (6.1–8.4)	7.8 (6.7–8.8)	8.6 (7.3–9.5)	<0.05 *^,†^
Behenic acid (22:0)	18.1 (14.6–20.5)	19.1 (16.4–21.9)	21.2 (18.3–23.8)	<0.05 ^†,‡^
Lignoceric acid (24:0)	16.0 (12.8–18.2)	15.9 (14.6–18.9)	18.6 (15.2–20.6)	<0.05 ^†^
Monounsaturated fatty acid				
Myristoleic acid (14:1n5)	0.8 (0.1–1.5)	1.3 (0.1–1.8)	1.4 (0.1–2.3)	<0.05 ^†^
Palmitoleic acid (16:1n7)	65.8 (53.6–91.6)	75.9 (56.2–111.9)	90.1 (76.6–127.9)	<0.05 ^†,‡^
Oleic acid (18:1n9)	654.0 (556.1–757.9)	714.0 (588.9–885.4)	807.1 (686.9–993.6)	<0.05 ^†,‡^
Eicosenoic acid (20:1n9)	4.7 (3.8–5.9)	5.4 (4.4–6.4)	5.5 (4.6–6.6)	<0.05 ^†^
Erucic acid (22:1n9)	1.1 (1.0–1.6)	1.1 (1.0–1.6)	1.3 (1.0–1.6)	
Nervonic acid (24:1n9)	35.9 (31.2–41.3)	35.2 (31.1–39.9)	37.4 (32.3–42.1)	
Polyunsaturated fatty acid				
Linoleic acid (18:2n6)	755.2 (670.4–869.5)	782.8 (672.4–910.1)	871.4 (772.7–1020.1)	<0.05 ^†,‡^
γ-linolenic acid (18:3n6)	8.7 (6.3–13.0)	10.8 (7.5–14.6)	11.2 (9.3–18.3)	<0.05 ^†^
α-linolenic acid (18:3n3)	20.2 (16.1–25.6)	21.7 (17.0–29.3)	25.5 (19.5–32.6)	<0.05 ^†^
Eicosadienoic acid (20:2n6)	6.4 (5.2–7.5)	6.5 (5.4–7.9)	6.9 (5.9–8.2)	<0.05 ^†^
Mead acid (20:3n9)	2.3 (1.4–3.2)	2.9 (1.7–4.2)	32.9 (2.1–4.2)	<0.05 ^†^
Dihomo-γ-linolenic acid (20:3n6)	40.5 (33.5–49.7)	48.9 (37.8–58.2)	59.8 (46.8–67.8)	<0.05 *^,†,‡^
Arachidonic acid (20:4n6)	183.0 (142.8–220.0)	209.7 (162.5–241.0)	209.2 (183.2–240.6)	<0.05 ^†^
Eicosapentaenoic acid (20:5n3)	43.4 (30.1–72.1)	45.6 (28.0–64.4)	50.4 (37.0–71.4)	
Adrenic acid (22:4n6)	5.2 (4.0–6.2)	5.7 (4.6–7.6)	6.3 (4.7–7.9)	<0.05 ^†^
Docosapentaenoic acid (22:5n3)	19.3 (15.9–22.4)	19.7 (15.5–25.0)	22.2 (17.8–27.7)	<0.05 ^†^
Docosahexaenoic acid (22:6n3)	130.5 (108.8–172.1)	130.3 (111.2–163.4)	149.8 (126.6–187.5)	<0.05 ^†^

Data are presented as median (interquartile range) or as numbers. *p*-values were determined using the Tukey–Kramer test or the Dwass–Steel test. *p* < 0.05 was considered statistically significant. * <33% vs. 33–66%; ^†^ <33% vs. >66%; ^‡^ <33–66% vs. >66%.

**Table 8:** Plasma fatty acid composition (μg/mL) (lobular inflammation).

Lobular Inflammation (per 200× Field)	<2 Foci (*n* = 106)	2–4 Foci (*n* = 93)	≥5 Foci (*n* = 36)	*p*-Value
Saturated fatty acid				
Lauric acid (12:0)	1.4 (0.9–2.3)	1.9 (1.2–2.8)	2.2 (1.5–3.4)	<0.05 *^,†^
Myristic acid (14:0)	21.1 (17.1–32.3)	26.1 (20.1–34.9)	30.0 (24.3–37.9)	<0.05 *^,†^
Palmitic acid (16:0)	724.2 (637.8–859.4)	779.9 (662.8–940.2)	843.4 (712.4–1006.7)	<0.05 ^†^
Stearic acid (18:0)	218 (187.4–255.7)	230.1 (193.0–262.7)	228.5 (213.2–257.9)	
Arachidic acid (20:0)	8.2 (6.6–9.0)	7.8 (6.4–8.9)	7.5 (6.8–8.5)	
Behenic acid (22:0)	19.5 (17.1–22.0)	19.0 (15.4–21.9)	19.3 (16.9–21.4)	
Lignoceric acid (24:0)	17.0 (14.9–19.3)	15.6 (13.4–19.2)	16.3 (13.9–18.8)	
Monounsaturated fatty acid				
Myristoleic acid (14:1n5)	0.8 (0.1–1.7)	1.1 (0.1–1.8)	1.5 (0.1–2.7)	
Palmitoleic acid (16:1n7)	71.6 (51.7–95.8)	77.6 (60.9–113.9)	96.6 (72.2–126.7)	<0.05 ^†^
Oleic acid (18:1n9)	673.0 (563.7–823.5)	728.3 (610.0–884.5)	783.7 (663.2–963.8)	<0.05 ^†^
Eicosenoic acid (20:1n9)	4.7 (3.9–6.3)	5.3 (4.5–6.3)	5.6 (4.5–6.5)	
Erucic acid (22:1n9)	1.0 (1.2–1.6)	1.2 (1.0–1.6)	1.2 (1.0–1.5)	
Nervonic acid (24:1n9)	36.9 (32.3–41.9)	35.4 (31.5–40.5)	35.8 (29.1–42.5)	
Polyunsaturated fatty acid				
Linoleic acid (18:2n6)	799.8 (682.2–938.1)	812.4 (705.2–969.7)	825.9 (728.7–978.6)	
γ-linolenic acid (18:3n6)	10.5 (7.3–15.1)	9.9 (7.0–13.4)	11.1 (8.7–16.4)	
α-linolenic acid (18:3n3)	21.5 (16.2–26.7)	21.8 (17.2–29.8)	25.1 (19.5–32.7)	
Eicosadienoic acid (20:2n6)	6.3 (5.1–7.5)	6.6 (5.5–8.1)	6.8 (6.1–7.9)	<0.05 ^†^
Mead acid (20:3n9)	2.6 (1.5–4.0)	2.5 (1.8–3.4)	3.1 (2.2–4.3)	
Dihomo-γ-linolenic acid (20:3n6)	45.3 (34.3–54.7)	48.4 (36.1–63.5)	53.8 (46.9–64.7)	<0.05 ^†^
Arachidonic acid (20:4n6)	199.5 (160.6–244.1)	192.8 (162.6–228.0)	199.8 (176.9–235.1)	
Eicosapentaenoic acid (20:5n3)	52.1 (33.4–74.0)	43.1 (28.5–66.6)	47.8 (27.6–63.2)	
Adrenic acid (22:4n6)	5.5 (4.3–7.2)	5.4 (4.5–6.9)	6.9 (5.4–7.9)	<0.05 ^†,‡^
Docosapentaenoic acid (22:5n3)	19.7 (15.8–24.4)	19.2 (16.0–24.8)	21.0 (18.2–26.5)	
Docosahexaenoic acid (22:6n3)	142.2 (115.5–179.7)	134.9 (111.7–171.0)	143.2 (108.8–166.6)	

Data are presented as median (interquartile range) or as numbers. *p*-values were determined using the Tukey–Kramer test or the Dwass–Steel test. *p* < 0.05 was considered statistically significant. * <2 foci vs. 2–4 foci; ^†^ <2 foci vs. ≥5 foci; ^‡^ 2–4 foci vs. ≥5 foci.

**Table 9:** Plasma fatty acid composition (μg/mL) (ballooning).

	None (*n* = 66)	Few Balloon Cells (*n* = 87)	Many Cells/Prominent Ballooning (*n* = 82)	*p*-Value
Saturated fatty acid				
Lauric acid (12:0)	1.4 (0.9–2.0)	1.7 (1.1–2.5)	2.2 (1.4–3.2)	<0.05 *^,†^
Myristic acid (14:0)	20.7 (16.0–31.2)	22.7 (18.1–31.3)	31.1 (23.5–38.9)	<0.05 *^,†^
Palmitic acid (16:0)	714.9 (627.1–801.7)	748.5 (633.0–883.6)	843.4 (727.6–1011.3)	<0.05 *^,†^
Stearic acid (18:0)	213.4 (187.0–255.8)	224.3 (187.4–258.1)	232.5 (208.3–263.6)	<0.05 ^†^
Arachidic acid (20:0)	8.0 (6.6–9.1)	7.8 (6.6–8.9)	7.9 (6.6–8.7)	
Behenic acid (22:0)	19.7 (16.7–22.6)	19.5 (16.8–21.9)	18.6 (15.9–21.3)	
Lignoceric acid (24:0)	16.8 (15.1–20.1)	16.3 (14.4–19.3)	16.0 (13.3–18.9)	
Monounsaturated fatty acid				
Myristoleic acid (14:1n5)	0.8 (0.1–1.5)	1.0 (0.1–1.7)	1.5 (0.1–2.6)	<0.05 *^,†^
Palmitoleic acid (16:1n7)	65.3 (48.8–89.9)	72.1 (56.6–90.3)	95.7 (71.7–128.6)	<0.05 *^,†^
Oleic acid (18:1n9)	654.0 (569.2–828.4)	679.3 (574.1–810.2)	775.2 (662.2–979.9)	<0.05 *^,†^
Eicosenoic acid (20:1n9)	4.7 (3.8–6.4)	5.1 (4.4–5.9)	5.2 (4.4–6.6)	
Erucic acid (22:1n9)	1.1 (1.0–1.5)	1.2 (1.0–1.7)	1.2 (1.0–1.5)	
Nervonic acid (24:1n9)	37.2 (31.7–42.4)	36.4 (31.8–40.2)	34.0 (31.2–41.8)	
Polyunsaturated fatty acid				
Linoleic acid (18:2n6)	799.8 (718.0–918.1)	809.3 (683.2–959.0)	824.3 (720.2–953.3)	
γ-linolenic acid (18:3n6)	11.0 (6.7–15.9)	9.7 (7.3–12.5)	10.9 (8.0–15.4)	
α-linolenic acid (18:3n3)	21.9 (17.6–27.2)	20.1 (16.4–27.8)	22.7 (18.2–29.8)	
Eicosadienoic acid (20:2n6)	6.2 (5.0–7.3)	6.4 (5.4–7.5)	6.9 (6.0–8.2)	<0.05 *^,†^
Mead acid (20:3n9)	2.3 (1.5–3.7)	2.4 (1.7–3.4)	3.1 (2.0–4.5)	<0.05 *^,†^
Dihomo-γ-linolenic acid (20:3n6)	44.6 (32.8–57.3)	44.6 (35.4–57.9)	52.6 (44.1–63.9)	<0.05 *^,†^
Arachidonic acid (20:4n6)	199.5 (175.0–247.1)	186.8 (157.9–230.1)	207.2 (167.7–235.4)	
Eicosapentaenoic acid (20:5n3)	47.4 (33.4–67.0)	50.0 (29.4–70.7)	43.2 (27.6–70.2)	
Adrenic acid (22:4n6)	5.3 (4.5–7.0)	5.4 (4.2–6.7)	6.3 (5–7.7)	<0.05 ^†^
Docosapentaenoic acid (22:5n3)	19.6 (15.2–24.5)	19.3 (15.6–24.3)	20.5 (17.8–26.5)	
Docosahexaenoic acid (22:6n3)	133.3 (110.5–170.9)	140.1 (116.4–176.9)	139.6 (107.4–168.8)	

Data are presented as median (interquartile range) or as numbers. *p*-values were determined using the Tukey–Kramer test or the Dwass–Steel test. *p* < 0.05 was considered statistically significant. * none vs. ≥5 foci; ^†^ few balloon cells vs. many cells/prominent ballooning.

**Table 10:** Risk of the fatty acids for advanced fibrosis (fibrosis stage 3–4).

	Crude HR (95% CI)	*p*-Value	Adjusted HR ^a^ (95% CI)	*p*-Value
Saturated fatty acid				
Lauric acid (12:0)	1.11 (0.95–1.32)	0.21		
Myristic acid (14:0)	1.003 (0.98–1.02)	0.74		
Palmitic acid (16:0)	0.99999 (0.999–1.001)	0.99		
Stearic acid (18:0)	1.25 (1.0002–1.58)	0.0498	1.001 (0.990–1.013)	0.84
Arachidic acid (20:0)	0.69 (0.58–0.82)	<0.01	0.60 (0.45–0.79)	<0.01
Behenic acid (22:0)	0.88 (0.82–0.94)	<0.01	0.86 (0.76–0.97)	0.01
Lignoceric acid (24:0)	0.90 (0.83–0.96)	<0.01	0.91 (0.80–1.03)	0.12
Monounsaturated fatty acid				
Myristoleic acid (14:1n5)	1.25 (1.0002–1.58)	0.0498	0.40 (0.04–3.95)	0.46
Palmitoleic acid (16:1n7)	1.006 (0.9998–1.0126)	0.06		
Oleic acid (18:1n9)	1.00003 (0.999–1.001)	0.95		
Eicosenoic acid (20:1n9)	1.01 (0.89–1.13)	0.92		
Erucic acid (22:1n9)	0.66 (0.33–1.27)	0.22		
Nervonic acid (24:1n9)	0.97 (0.94–1.01)	0.11		
Polyunsaturated fatty acid				
Linoleic acid (18:2n6)	0.998 (0.997–0.9998)	0.02	0.39 (0.04–3.93)	0.91
γ-linolenic acid (18:3n6)	0.97 (0.92–1.01)	0.17		
α-linolenic acid (18:3n3)	0.97 (0.94–0.99)	0.01	0.92 (0.87–0.97)	<0.01
Eicosadienoic acid (20:2n6)	0.98 (0.84–1.12)	0.74		
Mead acid (20:3n9)	1.16 (1.01–1.34)	0.04	1.34 (1.10–1.65)	<0.01
Dihomo-γ-linolenic acid (20:3n6)	0.998 (0.98–1.01)	0.79		
Arachidonic acid (20:4n6)	0.994 (0.988–0.998)	<0.01	0.994 (0.988–1.001)	0.11
Eicosapentaenoic acid (20:5n3)	0.987 (0.977–0.996)	<0.01	0.97 (0.96–0.98)	<0.01
Adrenic acid (22:4n6)	1.03 (0.92–1.15)	0.60		
Docosapentaenoic acid (22:5n3)	0.96 (0.92–0.99)	0.01	0/89 (0.83–0.95)	<0.01
Docosahexaenoic acid (22:6n3)	0.989 (0.982–0.994)	< 0.01	0.98 (0.97–0.99)	<0.01

^a^ Multivariate Cox proportional hazards regression analysis adjusted for age, sex, BMI (kg/m^2^), ALT (U/L), HbA1c (%), Cre (mg/dL), TC (mg/dL), TG (mg/dL), and NAS. Differences were considered statistically significant at *p* < 0.05. HR, hazard ratio; CI, confidence interval; BMI, body mass index; ALT, alanine aminotransferase; HbA_1c_, hemoglobin A_1c_; Cre, creatinine; TC, total cholesterol; TG, triglycerides; NAS, NAFLD Activity Score.

**Table 11:** Risk of the fatty acids for high NAS.

	Crude HR (95% CI)	*p*-Value	Adjusted HR ^a^ (95% CI)	*p*-Value
Saturated fatty acid				
Lauric acid (12:0)	1.56 (1.25–1.99)	<0.01	1.23 (0.91–1.70)	<0.01
Myristic acid (14:0)	1.07 (1.04–1.10)	<0.01	1.07 (1.01–1.13)	0.01
Palmitic acid (16:0)	1.004 (1.003–1.006)	<0.01	1.007 (1.003–1.013)	<0.01
Stearic acid (18:0)	1.013 (1.008–1.020)	<0.01	1.01 (0.99–1.02)	0.36
Arachidic acid (20:0)	1.22 (1.04–1.43)	0.01	1.10 (0.82–1.49)	0.52
Behenic acid (22:0)	1.06 (0.99–1.12)	0.08		
Lignoceric acid (24:0)	1.03 (0.96–1.10)	0.44		
Monounsaturated fatty acid				
Myristoleic acid (14:1n5)	1.49 (1.18–1.93)	<0.01	1.30 (0.89–1.91)	0.17
Palmitoleic acid (16:1n7)	1.024 (1.016–1.035)	<0.01	1.015 (1.003–1.030)	0.02
Oleic acid (18:1n9)	1.004 (1.002–1.005)	<0.01	1.006 (1.001–1.012)	0.01
Eicosenoic acid (20:1n9)	1.14 (1.01–1.31)	0.03	0.93 (0.75–1.14)	0.49
Erucic acid (22:1n9)	1.57 (0.84–3.04)	0.16		
Nervonic acid (24:1n9)	1.01 (0.98–1.05)	0.54		
Polyunsaturated fatty acid				
Linoleic acid (18:2n6)	1.002 (1.001–1.003)	<0.01	1.001 (0.998–1.004)	0.37
γ-linolenic acid (18:3n6)	1.041 (0.995–1.090)	0.08		
α-linolenic acid (18:3n3)	1.03 (1.01–1.07)	0.01	1.0005 (0.9424–1.0628)	0.99
Eicosadienoic acid (20:2n6)	1.30 (1.12–1.54)	<0.01	1.05 (0.81–1.35)	0.73
Mead acid (20:3n9)	1.32 (1.13–1.57)	<0.01	1.09 (0.86–1.39)	0.50
Dihomo-γ-linolenic acid (20:3n6)	1.05 (1.03–1.07)	<0.07	1.02 (0.99–1.06)	0.13
Arachidonic acid (20:4n6)	1.006 (1.001–1.011)	<0.01	1.001 (0.993–1.009)	0.75
Eicosapentaenoic acid (20:5n3)	0.996 (0.989–1.004)	0.35		
Adrenic acid (22:4n6)	1.31 (1.14–1.52)	<0.01	1.21 (0.95–1.56)	0.12
Docosapentaenoic acid (22:5n3)	1.05 (1.01–1.09)	<0.01	1.01 (0.95–1.08)	0.66
Docosahexaenoic acid (22:6n3)	1.003 (0.998–1.008)	0.24		

^a^ Multivariate Cox proportional hazards regression analysis adjusted for age, sex, BMI (kg/m^2^), ALT (U/L), HbA1c (%), Cre (mg/dL), TC (mg/dL), TG (mg/dL), and fibrosis stage. Differences were considered statistically significant at *p* < 0.05. NAS, NAFLD Activity Score; HR, hazard ratio; CI, confidence interval; BMI, body mass index; ALT, alanine aminotransferase; HbA_1c_, hemoglobin A_1c_; Cre, creatinine; TC, total cholesterol; TG, triglycerides.

## Data Availability

The datasets used and/or analyzed during the current study are available from the corresponding author on reasonable request.
